# A Hemostatic Technique in Robot-Assisted Laparoscopic Partial Nephrectomy and Its Impact on Renal Function

**DOI:** 10.7759/cureus.15122

**Published:** 2021-05-19

**Authors:** Deepak Raghavan, Mathisekaran Thangarasu, Sanjay Prakash J, Rajesh Paul, Nivash Selvaraj

**Affiliations:** 1 Urology, Apollo Hospitals, Chennai, IND

**Keywords:** partial nephrectomy, tumor, hemostatic, complications, renal function

## Abstract

Purpose

Robot-assisted partial nephrectomy (RAPN) has become popular in recent years for small renal masses. We describe a technique of suturing renal defects during RAPN that is reliable and quick, does not necessitate the need for hemostatic agents, and reduces perioperative complications.

Materials and methods

A total of 24 patients who underwent RAPN were included in the study period between 2013 and 2018 and data were analyzed. Perioperative and postoperative outcomes were measured and compared.

Results

The median tumor size was 4 cm. Median warm ischemia time was 41 minutes (IQR: 38-45 minutes) and estimated blood loss was 150 mL (IQR: 120-200 mL). There were no major intraoperative complications or conversions to open surgery. No urine leaks or postoperative bleedings were observed.

Conclusion

Our technique is safe and effective. It negates the use of hemostatic agents, decreases perioperative complications, and negates that determination of long-term renal function is not associated with prolonged warm ischemia time alone. Hence, we propose that our technique is safe in partial nephrectomy when the pelvic calyceal system and renal vessels are opened in multiple locations.

## Introduction

Renal cell carcinoma is the commonest cancer seen in the genitourinary department. With the increased incidence of diagnosing small renal masses, urologists are motivated to improve the contemporary techniques [1]. Currently, small masses of kidneys are well managed by partial nephrectomy (PN) as a substitute for radical nephrectomy. Presently, robot-assisted partial nephrectomy (RAPN) is becoming the standard of care for renal masses, which has its own advantages such as 3D vision, better ergonomics, lower morbidity, reduced postoperative complications, and shorter hospital stay. However, the renal defect closure and control of bleeding under pressure of a ticking clock remains a common problem. We describe a technique in RAPN which minimizes the devascularization of parenchyma without any need of hemostatic agents and preserves the maximum healthy renal parenchyma and fewer complications.

## Materials and methods

This study was approved by the Institutional Ethics Committee - Clinical Studies, Apollo Hospitals, Chennai, India, and was conducted between January 2013 and March 2018. The written informed consent was obtained from the patients prior to the procedure. A total of 24 patients underwent RAPN. All the procedures were performed by a single experienced robotic surgeon. All the patients underwent preoperative workup, which included serum creatinine, estimated glomerular filtration rate (eGFR), and radiological assessment by contrast-enhanced triphasic CT for nephrometric score. The measured outcomes were operative time, warm ischemia time (WIT), estimated blood loss, margin status, and postoperative complications. The patients were followed up for 24 months postoperatively for the renal function and cancer status. Serum creatinine and eGFR were used to assess the renal function during the follow-up period. The Modification of Diet in Renal Disease (MDRD) study equation was used to assess the renal status of the patient [2].

Statistical analysis

The data were statistically analyzed using SPSS 20.0 Version (IBM Corp., Armonk, NY). The non-normally distributed variables are presented as the median. Nominal data are presented as a number or percentage and compared using the chi-square test. Differences were compared between the follow-up period and the baseline. A p-value of less than 0.05 was considered statistically significant.

Surgical technique

Under general anesthesia and antibiotic coverage, the patient was in lateral decubitus position with parts painted and draped. No prior double J stenting was performed in any patients. Pneumoperitoneum was created, and the standard five- or six-port insertion technique was followed (1-12 mm or 4-8 mm based on the side). The DaVinci Xi Surgical System (Intuitive Surgical Inc., Sunnyvale, CA) was docked. The following steps were involved:

Step 1: The colon was mobilized and dropped down.

Step 2: The gonadal vein and ureter bundle were identified and independently preserved, and dissection proceeded towards the hilum.

Step 3: The hilar vessels were skeletonized, the renal artery as well as the renal vein were looped, and dissection was performed in extra gerota’s fascia.

Step 4: Gerota’s fascia overlying the tumor was opened and the tumor was visualized. Intra-operative ultrasound was used to ascertain tumor margins and depth.

Step 5: Renal arteries were clamped with vascular Scanlan® Bulldog Clamps (Scanlan International, Saint Paul, MN).

**Figure 1:**
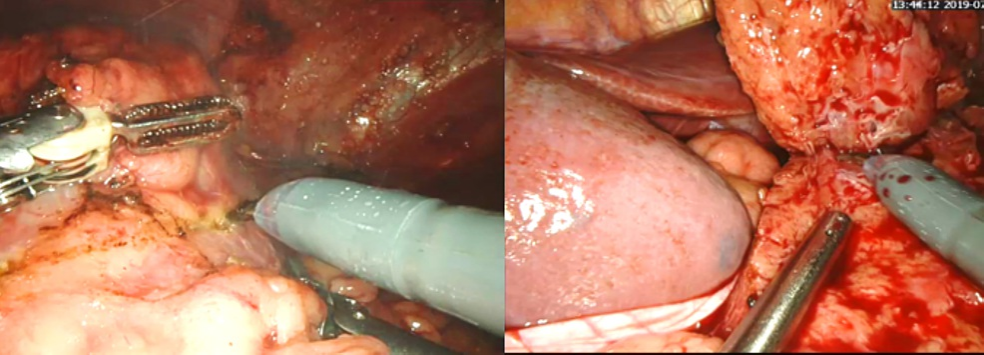
Tumor scoring and excision

Step 6: Scoring of the tumor was done, and the tumor was dissected with healthy tissue margin (Figure 1).

**Figure 2:**
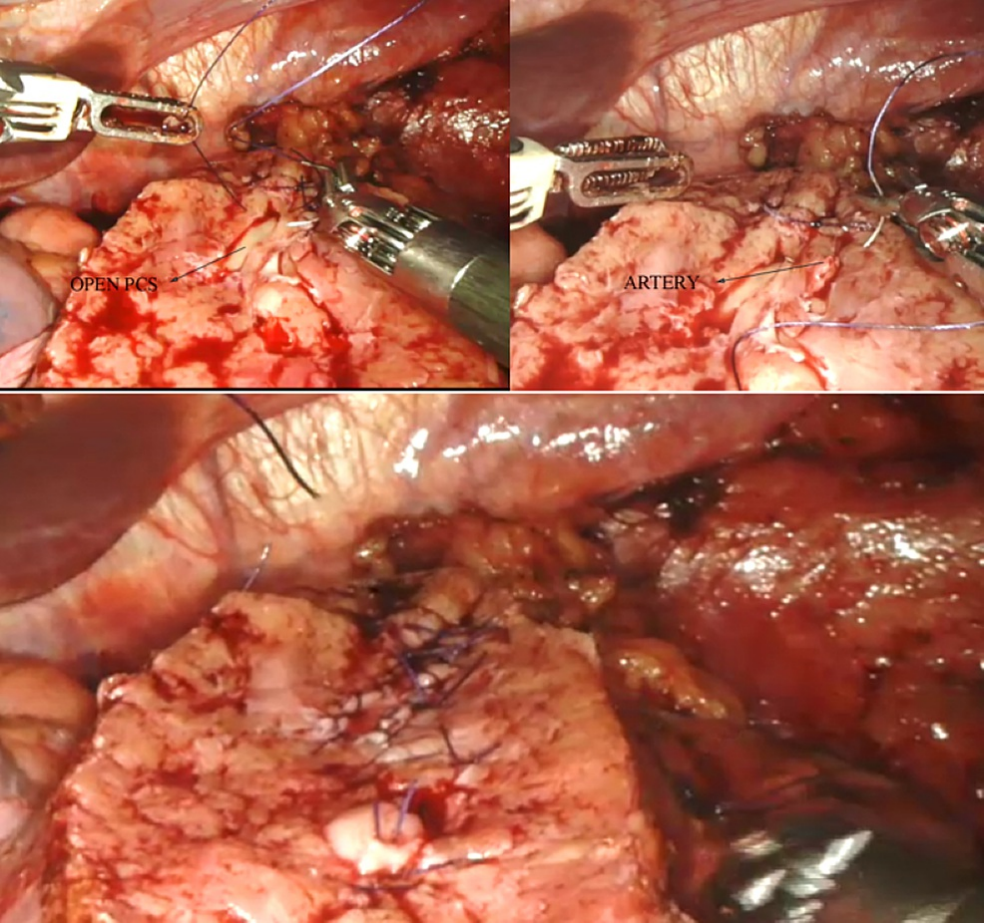
Completion of individual renal vessels ligation and closure of the opened pelvic calyceal system

**Figure 3:**
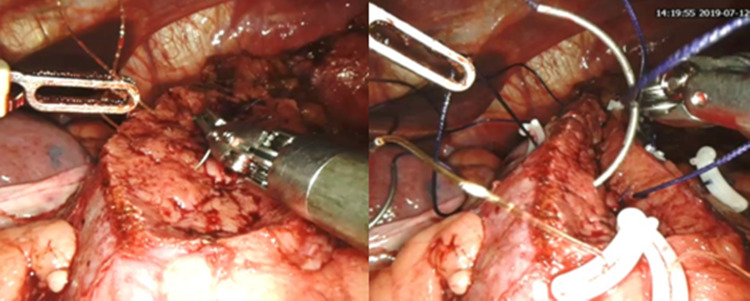
Completion of the inner corticomedullary junction by continuous suture and outer renorraphy with interrupted layer

In the next step (step 7), the exposed individual vessels were ligated at the resected renal parenchyma and tumor base with 4-0 Vicryl suture (Ethicon, Somerville, NJ) using a figure-of-eight technique (Figure 2). If the pelvic calyceal system (PCS) was opened, it was meticulously closed with 4-0 Vicryl suture in a continuous manner. The cortex and corticomedullary junction were closed with continuous sutures as an inner layer renorrhaphy with 2-0 Stratafix™ (Ethicon). Interrupted sliding clip outer layer renorrhaphy was performed with 1-0 Vicryl suture (Figure 3). Finally, vascular clamps were released and a flat drain was placed. The specimen was bagged and retrieved via incision in the iliac fossa. Wound was closed in layers, and skin was sutured with 3-0 Monocryl suture (Ethicon).

 

## Results

All the patients successfully completed the study. The median age of the patient was 50 years. Preoperatively four patients were having chronic kidney disease (CKD) stage ≥ 3 disease. The median preoperative creatinine and eGFR were 0.95 mg/dL and 90 mL/min/1.73m^2^, respectively. The demographic profile of the patient is given in Table 1.

**Table 1:** Demographic profile of the patient (n=24) ASA, American Society of Anesthesiologists; BMI, body mass index; CKD, chronic kidney disease; eGFR, estimated glomerular filtration rate; IQR, interquartile range *eGFR < 60 mL/min/1.73m^2^

Characteristics	
Age (in years), median (IQR)	50 (17-59)
Gender % (male/female)	20 (83%)/4 (17%)
BMI, median (IQR)	24.7 (22.9-27.1)
ASA grade (1/2/3/4)	2/6/14/2
CKD stage ≥ 3 (%)^*^	4 (17%)
Preoperative creatinine (mg/dL), median (IQR)	0.95 (0.8-1.2)
Preoperative eGFR (mL/min/1.73m^2^), median (IQR)	90 (66-101)
Laterality (right/left), %	11/13 (46%/54%)
Clinical stage	
T1a, n	17
T1b, n	7
Site	
Upper, n	8
Interpole, n	6
Lower, n	10
Renal score, median (IQR)	5 (4-6)

There was no conversion to open surgery. The median operative time was 130 minutes (IQR: 108-160 minutes), and the median WIT was 41 minutes (IQR: 38-45 minutes) and for this step it was 3 minutes. There were no urine leak or major intraoperative and postoperative complications recorded in our study. The median estimated blood loss was 150 mL (IQR: 120-200 mL), and the median length of stay was four days. All patients were having zero positive margins. The overall perioperative outcomes are shown in Table 2.

**Table 2:** Overall perioperative outcome IQR, interquartile range

Variables	Median (IQR)
Operative time (minutes)	130 (108-160)
Estimated blood loss (mL)	150 (120-200)
Warm ischemic time (minutes)	41 (38-45)
Margin positivity	Nil
Pathological diagnosis	
Benign	2
Malignant	22
Length of stay (days)	4 (3-5)

Moreover, the median serum creatinine levels were significantly increased and eGFR was significantly decreased after RAPN. However, no significant differences were observed between the preoperative values after one-year and two-year follow-up. Tables 3, 4 show information about the renal function changes over time.

**Table 3:** Renal function changes over time P1: statistical difference between preoperative creatinine value and day 2 creatinine value. P2: statistical difference between preoperative creatinine value and three-month creatinine value. P3: statistical difference between preoperative creatinine value and six-month creatinine value. eGFR, estimated glomerular filtration rate; IQR, interquartile range

Parameters	Preoperative value	Day 2	Three months	Six months	P1	P2	P3
Creatinine, median (IQR)	0.9 (0.8-1.2)	1.5 (1.3-1.5)	2 (1.3-2.6)	1.5 (1.2-2.2)	0.01	0.014	0.02
eGFR (mL/min/1.73m^2^), median (IQR)	90 (67-102)	49 (36-63)	37 (30-57)	50 (29-68)	0.002	0.01	0.04

**Table 4:** Renal function changes over time P1: statistical difference between preoperative creatinine value and one-year creatinine value. P2: statistical difference between preoperative creatinine value and two-year creatinine value eGFR, estimated glomerular filtration rate; IQR, interquartile range

Parameters	Preoperative value	One year	Two years	P1	P2
Creatinine, median (IQR)	0.9 (0.8-1.2)	1.2 (1.1-1.5)	1.2 (1.1-1.5)	0.39	0.65
eGFR (mL/min/1.73m^2^), median (IQR)	90 (67-102)	63 (45-73)	61 (50-78)	0.47	0.32

## Discussion

RARP is the standard of care for most of the patients with T1 renal lesions in many tertiary centers. The renal function preservation is the primary aim in PN and can be done by open, laparoscopic, or robotic methods [3]. The various factors that predict renal functional status after PN include tumor, patient, and surgery-related factors, out of which the only factor that can be optimized is the surgery-related factor [4].

The clamping of renal vessels in PNs is generally needed to manage blood loss, attain a negative-positive margin, and execute precise renorrhaphy. Numerous techniques have been documented in the literature with the aim of preserving renal parenchyma and have fewer complications [5-7]. The present study describes a surgical technique in which the renal vasculature is preserved and parenchymal devascularization is minimized with no major postoperative complications.

In the present study, the median warm ischemic time was 41 minutes (IQR: 38-45 minutes). This was in contrast with many other studies. However, several studies have shown that the final renal function of the kidney subjected to PN correlated primarily with the preserved parenchyma volume, whereas only a secondary role was played by WIT, irrespective of whether WIT was achieved by flow restriction or hypothermia [8]. Recently, Nahar et al. reported that in PN, baseline renal function and the amount of preserved parenchyma play a major role in determining the final outcome. The limited duration of ischemia does not have a significant role impact on final renal function [9]. Moreover, the focus should rather be on appropriate perioperative management of patients, which includes controlling diabetes and hypertension, identifying proteinuria and CKD, preservation of renal parenchyma volume, and timely referral to nephrologists for optimization postoperatively. In our study, even though the serum creatinine was significantly increased and eGFR was significantly decreased, at two-year follow-up, we did not encounter deterioration in renal function.

In our study, the technique of independently suturing opened blood vessels and the opened PCS with Vicryl before performing the two-layer renorrhaphy resulted in a median blood loss of 150 mL. No significant bleeding was encountered upon clamp release intraoperatively or postoperatively. In addition, Seideman et al. showed reduced incidence of major intraoperative bleeding by using barbed suture [10]. Similar outcomes were documented in other series using V-Loc™ (Medtronic, Minneapolis, MN) [11-12]. The results of the present study were in concordance with the previous studies.

Omae et al. reported an unanticipated increasing rate of about 21.7% for unruptured asymptomatic pseudoaneurysm of the renal artery, as identified by CT arteriography [13]. In addition, Jain et al. in a systematic and comparative analysis reported symptomatic pseudoaneurysm in 1.96% of cases [14]. In this present study, we did not encounter any such incident during the postoperative follow-up. One of the main limitations of this study is the lack of a control group and small sample size with low complexity.

## Conclusions

Thus, our technique is simple and effective in reducing perioperative complications and dealing with difficult anatomy. It also negates the use of hemostatic agents and that determination of long-term renal function is not associated with prolonged WIT alone. Hence, we propose that our technique is safe in PN when PCS and renal vessels are opened in multiple locations. However, further randomized control studies with lengthier follow-up are needed.
